# No impact of a massive transfusion protocol on coagulopathy and mortality at a level 1 trauma center: why?

**DOI:** 10.1186/cc11051

**Published:** 2012-03-20

**Authors:** C Bourassa-Fulop, J Chauny, J Paquet, R Daoust, E Notebaert

**Affiliations:** 1 Hôpital Sacré-Coeur, Montreal, Canada

## Introduction

In 2010 we studied the mortality and coagulopathy of all massively transfused patients at our hospital since 2004. We compared those who were transfused before the implementation of our massive transfusion protocol (MTP) (from 2004 to 2006) to those transfused with MTP. We found that our MTP did not lower mortality (35.7%) and our incidence of coagulopathy was high (72.6%). The aim of the present study is to explain those results, while concentrating uniquely on trauma patients.

## Methods

We conducted a retrospective nested case-control study from our trauma registry. We included trauma patients who received 10 packed red blood cells (pRBC) or more in 24 hours and excluded those who died within the very first hours of massive trauma. We extracted supplementary demographic and clinical data from the laboratory database and the hospital files. Chi-square tests and multivariate logistic regression were used to compare the effect of the two approaches (MTP vs. non-MTP) on mortality and coagulopathy, defined as an INR ≥1.8, a PTT ≥54, a fibrinogen <1 g/l or a platelet count <50,000, while controlling for acidosis (defined as a pH ≤7.1), hypothermia (defined as ≤35°C) and Injury Severity Score (ISS) (critically injured if ISS ≥30).

## Results

Of the 84 trauma patients, 23 were transfused with the MTP and 61 without. The average ISS score was very high (29.2), most were male (73.8%) and the average age was 41 years. The MTP versus non-MTP groups were similar in regards to age, sex, pH, temperature, ISS and Revised Trauma Score, but the MTP group received more transfusions (40% vs. 22% when dichotomized in two groups: above 20 pRBC and between 10 and 20 pRBC). The mortality and coagulopathy were similar in both the MTP and non-MTP group (39% vs. 34% and 65% vs. 75% respectively). PTM did not affect mortality or coagulopathy, even when controlling for all other variables. Individually, both hypothermia (OR = 2.6, 95% CI: 1.1 to 6.8) and acidosis (OR = 4.3, 95% CI:1.6 to 13.0) significantly affected mortality, while the number of pRBC (OR = 3.8, 95% CI: 1.1 to 14.1) was the main determinate for coagulopathy.

## Conclusion

In our population of severely injured patients, the MTP was not found to be beneficial in regards to mortality nor coagulopathy. Hypothermia and acidosis seem to be the main determinants for mortality and should be among the priorities in caring for trauma patients.
